# Cross-sectional survey of risk factors for edema disease *Escherichia coli* (EDEC) on commercial pig farms in Germany

**DOI:** 10.1186/s12917-025-05054-7

**Published:** 2025-10-03

**Authors:** Pia I. Berger, Steffen Hermanns, Friederike Schmelz, Katharina Kerner, Daniel Sperling, Christa Ewers, Rolf Bauerfeind, Marcus G. Doherr

**Affiliations:** 1 Institute of Veterinary Epidemiology and Biostatistics, Freie Universität Berlin, Berlin, Germany; 2 Institute for Hygiene and Infectious Diseases of Animals, Justus Liebig University, Giessen, Germany; 3 IDT Biologika GmbH, Dessau-Rosslau, Germany; 4 Ceva Santé Animale, Libourne, France

**Keywords:** Edema disease, Pigs, EDEC, *Escherichia coli*, Stx2e, Logistic regression, GLM

## Abstract

**Background:**

Edema disease (ED) in swine, usually caused by certain Shiga toxin-producing *Escherichia coli* (EDEC) in the first few weeks after weaning, has a high mortality rate in affected piglets and can lead to high economic losses in rearing. In the published first part of the study, we found EDEC in 37.4% of all investigated farms keeping weaned piglets in Germany [1]. In this part of the project, we analyzed risk factors for the presence of EDEC on those farms by using an interview-based questionnaire.

**Results:**

During the interview, data on farm structure and performance, health status of weaned piglets, farm management as well as feeding and water supply were collected from the farm managers. Univariable analyses using either cross tabulation and a 2-sided Fisher´s exact test (FET) or a one-way analysis of variance (ANOVA) identified factors potentially associated with farm-level EDEC presence. Multivariable logistic regression models (outcome: farm positive for EDEC) as well as negative binomial regression models (outcome: number of pens per farm positive for EDEC) were used to identify final risk factors. Higher age of piglets at the beginning of pre-starter feeding (in the farrowing area) increased the EDEC risk. Significantly, the risk increased for those farms that did not provide a pre-starter to the piglets until after day ten of life (odds ratio 4.64; p-value 0.015). The use of certain vaccines (STEC, *Lawsonia intracellularis* and *Clostridium* spp.) also yielded significant results. Farms with higher weaning weights and higher weights at the end of the flat deck period had a higher risk of EDEC presence, whereas the results in relation to weaning age were inconclusive.

**Conclusions:**

Many variables that are considered risk factors for ED have already been excluded due to lack of significance after univariable analysis. Nevertheless, early feeding of a pre-starter in the farrowing area seems to reduce the risk of detecting EDEC in weaned piglets, as well as it seems beneficial for farms to vaccinate sows against *Clostridium* spp. Our study also showed a higher risk of EDEC detection for farms with superior performance/ high piglet weights. The influence of the weaning age may be the subject of further investigations.

**Supplementary Information:**

The online version contains supplementary material available at 10.1186/s12917-025-05054-7.

## Background

The period after weaning is a critical phase in the rearing of piglets, as animals at this age are particularly susceptible to infectious diseases [2]. One of them is edema disease (ED), a potentially lethal disease of weaned piglets worldwide [3–8]. The causative agent, so-called edema disease *Escherichia coli* (EDEC), represents a porcine specific subgroup of Shiga toxin-producing *E. coli* (STEC). After oral uptake, EDEC multiply in the digestive tract of piglets and colonize the mucosa of the small intestine [9]. Adhesive proteinaceous projections on the bacterial surface, the F18 type fimbriae, mediate specific attachment of EDEC bacteria to epithelial cells of the intestinal mucosa [10–12]. Intestinal EDEC produce Shiga toxin subtype Stx2e (Stx2e) which enters the blood circulation of pigs and subsequently damages endothelial cells of the microvascular system. The damage is followed by fluid leakage and edema formation in various organs [13–15]. In addition to Stx2e and F18 fimbriae EDEC may also produce hemolysins and heat-labile and/or heat-stabile *E. coli* enterotoxins [16–18].

In case of acute disease progression, development of edema in the central nervous system (CNS) is fatal for the piglet within a few hours. Therapy in an acutely affected animal is rarely effective, and neither sensible from an economic point of view nor justified by animal welfare [19, 20]. The clinical signs of ED include high pitch vocalizations, dyspnea, and symptoms typical of CNS disorders, such as rowing movements in the lateral position [15].

Various strategies have been developed to reduce frequency and severity of clinical manifestations of ED. Effective protection against ED can be achieved using Stx2e toxoid vaccines in suckling piglets, which can reduce the economic losses resulting from animal deaths and treatment costs after weaning [21, 22]. Feed restriction, general optimization of husbandry conditions, and the use of antimicrobial agents and administration of zinc oxide at pharmacological levels have also been recommended [23–29]. However, development of antimicrobial resistance in STEC can be considered critical, since resistance to more than one antibiotic class has been detected in several studies [23, 29–32]. This phenomenon may not only be caused by extensive use of antibiotics, as there is evidence that also high dietary zinc supplementation can increase resistance and multidrug resistance in *E. coli* [23, 33–35]. Furthermore, treatment with antibacterial agents must be expected to result in a more rapid release of the Stx2e from killed EDEC bacteria in vivo, resulting in significantly more severe cases of ED and deaths from it [36, 37]. In summary, neither the use of zinc oxide nor the use of antibiotics should be preferred to immunization of piglets. However, vaccination does not meet with unanimous approval by all piglet producers as it is comparatively labor- and cost-intensive. Therefore, it seems logical and sensible to search for other starting points of preventive measures against ED in pig husbandry.

In a previous cross-sectional study on the prevalence of EDEC in weaned piglets in Germany, we found that 37.4% of the 99 farms studied had pens in which EDEC were detected by bacterial culture or by whole DNA extraction and subsequent PCR analysis [1]. In this second part of the study, we present the results of an interview-based survey on these farms which covered domains such as farm structure and performance, health status of weaned piglets, farm management including various hygiene measures and climate conditions as well as factors related to feeding and water supply. Generalized linear models (GLM) were used to identify those factors that were significantly associated with the presence of EDEC in pens containing weaned piglets on the study farms.

## Methods

### Study design

As part of a cross-sectional study, non-invasive sampling was conducted on pig farms in Germany, targeting pens with recently weaned piglets [1]. As previously described, during one-time visits samples had to be collected from five randomly selected pens per farm. Four sample types were collected concurrently in each of these pens: (a) five single fecal samples from the pen floor, (b) a boot swab sample from the pen floor, (c) an oral fluids sample using a non-invasive sampling technique (chewing rope offered to all piglets in a pen), and (d) a rope wash out sample. On each farm, a questionnaire on risk factors for edema disease was completed by the veterinarians together with the farm managers on site. Each farm was visited once for sampling, the first in December 2018 and the last in March 2020.

### Recruitment of participating pig farms

Given the limitations in time and personnel both at the level of farm visits but even more at the level of the laboratory responsible for testing pen-level samples from all sampling protocols and in different tests, it was decided to limit the number of farms to be recruited for the study to 100. Ninety-nine pig farms in Germany could eventually be recruited for the project through their herd veterinarian, which were called in person by the study researchers for recruitment. This resulted in a precision in the EDEC prevalence estimate of approximately ± 10% at farm level and ± 5% at pen level, which was considered sufficient to achieve the overall study objective. Participation in the project was voluntary, and farms were not reimbursed; however, they received the results of their laboratory tests free of charge. A reasonable farm size was part of the inclusion criteria to ensure that the farms – at the time of the visit – had at least five pens with piglets 1–2 weeks after weaning to be sampled. Additional effort was made to achieve a reasonable geographic coverage of the main swine producing regions in Germany. Prior to the visit, the farms were informed about the project objectives and the process of the farm visit.

### Sampling protocol at farm level and processing of samples

A detailed description of the sampling on the farms, as well as the processing of samples, can be found in our publication on the prevalence of EDEC in weaned piglets in Germany [1]. Briefly, all samples were examined systematically for putative *E. coli* bacteria by culture methods. Aliquots of boot swab samples, oral fluid samples, and rope wash out samples were also submitted to whole DNA extraction. Following this, suspected *E. coli* isolates and extracted total DNA samples were analyzed via PCR to detect the presence of the stx2e and fedA genes. A sample was considered positive for EDEC when *E. coli* encoding both for Stx2e and F18 fimbriae was isolated or when *stx2e* and *fedA* were simultaneously detected in the DNA extracted from that sample. A pen was classified as EDEC-positive if any of the collected samples tested positive, and farms were considered EDEC-positive if at least one pen on the premises was classified as positive.

### Questionnaire to assess risk factors

To evaluate possible risk factors for the presence of EDEC, a questionnaire was developed by authors of this study and pretested with a veterinary practitioner specialized in porcine heard health management. An effort was made to include those farm level factors that had been either shown or suggested in the available scientific literature as risk factors for ED in weaned piglets. Questionnaires were completed by the veterinarians during the farm visits in an interview with the respective farm managers. Within the questionnaire, information on farm structure, health status of the weaning piglets, hygiene management, stable climate, as well as feeding and water supply were requested systematically. The information on the current symptoms of weaned piglets was estimates made by the veterinarians and/or farm managers. The indications of previous detection of pathogens in the laboratory (STEC, *Escherichia coli*, TGEV, EVDV, Rotavirus, *Clostridium* spp., *Salmonella* spp., *Lawsonia intracellularis*, *Streptococcus* spp., *Glaesserella parasuis*, PCV-2, PRRSV, *Brachyspira hyodysenteriae/pilosicoli*, parasites or others), like all other data, were based on the records and information provided by the responsible veterinarians and farm managers. Questionnaire entries were transferred into an MS Excel worksheet, with each farm representing one row and therefore the unit of analysis. The full questionnaire (English translation) can be found in the supplementary material (Additional file 1).

### Statistical analyses

Data were evaluated using statistical software packages SPSS v25 and STATA v15. Questionnaire data compiled at farm level were merged with testing results received from the laboratory. Farm-level classification (positive or negative) for EDEC detection and the number of pens positive for EDEC per farm were defined as outcome variables. Frequencies and descriptive statistics were derived to identify the distribution of values by variable and category. Depending on the format and distribution of values, initial variables were transformed either numerically (such as a log_10_ transformation), or they were categorized based on biological or statistical (such as median or quartiles) cut-off values. After assessing the distribution of values, variables were selected for the intended risk factor models based on biological plausibility and sufficient variability in values (such as category frequencies > 5). Parameters with too small variability between farms and category frequencies of zero were excluded from further analysis in the subsequent regression model after the frequency analysis. All selected potential risk factors and their transformations were tested for their univariable association with farm-level EDEC status (coded 0 or 1) using either cross tabulation and a 2-sided Fisher´s exact test (FET) or a one-way analysis of variance (ANOVA). Those factors with a univariable p-value < 0.25 were considered candidates for the subsequent generalized linear model (GLM) approach. In case of several transformations of the same original variable, the variant that provided the most feasible interpretation was selected for further analysis by the study researchers following an expert discussion. For each domain, candidate variables that met the inclusion criteria were included into one of four multivariable logistic regression models (outcome: farm positive for EDEC) and four multivariable negative binomial regression models (outcome: number of pens per farm positive for EDEC, corrected by the number of pens tested) - one regression model of each for the domains (i) feeding and water supply, (ii) health, (iii) management and (iv) farm structure and performance. The generalized linear model (glm) function of STATA with family either binomial (and logit link) to generate OR parameter estimates or negative binomial (ang log link) to generate IRR parameter estimates were used. In each domain, initially three selection routines were employed: forward (p-to-enter 0.05), backward (p-to-remove 0.05) and backward stepwise (p-to-enter 0.05, p-to-remove 0.10). All variables maintained in one of the three approaches were included in a final model for that domain without further selection procedure. Parameter estimates (OR or IRR) with 95% CI were derived using the default maximum likelihood algorithm of STATA. Within each domain, confounding between variables in the final model between variables in the model was automatically controlled for. Further confounding effects of variables within and between domains were not assessed.

## Results

### Farm characteristics

In the first part of this cross-sectional study, we found that 37.4% of the 99 farms studied had pens in which EDEC were detected by bacterial culture or by whole DNA extraction and subsequent PCR analysis [1].

Six farms had to be excluded from further analysis due to missing entries in the questionnaires, leaving data from 93 study farms distributed across Germany to be analyzed. Most of these farms were engaged in conventional pig farming (87.1%), while only three were organic farms (3.2%). About half of the farms were piglet producers (50.5%). Farms worked in different production rhythms: a three-week batch management was the most frequently used rhythm (35.6% of the farms), followed by weekly weaning (25.6%). Farms could voluntarily indicate whether the occupancy of flat decks exceeded the legal requirements. This question was answered by 86 of the farms, of which 37.2% stated that they partially or notably overcrowded the flat deck area. Among the sampled farms in the study, 12.9% reported earlier lab detection of STEC and 16.5% of study farms reported vaccinating the piglets against STEC.

The health status of weaned piglets was reported as percentages of symptomatic animals by 92 farms in the study. Diarrhea was observed most frequently (average 12.3% of piglets), 3.6% of piglets suffered from dyspnea, 2.1% of piglets were retarded, 1.9% of piglets suffered from central nervous symptoms, while sudden death was observed in 1.3% of the piglets. Subcutaneous edema (average 1.0% of piglets) was the least common symptom reported.

The median age of piglets at weaning was 26 days, and piglets were moved to the flat deck with an average body weight of 7.07 kg. The piglets stayed there an average of 53.1 days before they were transferred to the fattening areas with a mean body weight of 28.4 kg. The average losses during stay in the flat deck area were reported to be 2.3%.

Additional descriptive information on participating farms is summarized in Additional Tables 1, 2, 3 and 4.

### Risk factors for presence of EDEC at farm level

Of the feeding and water supply-related variables in the questionnaire, 54 variables (including different transformations of some variables) were selected according to biological plausibility as risk factor candidates for further analysis after the frequencies and descriptive statistics had been calculated. These variables were analyzed for their association with farm level EDEC status in univariable models using ANOVA or Fisher’s exact test (depending on the format of the risk factor) (Additional Table 5).

In a second step, those seven variables that had an univariable p-value < 0.25 were included into the multivariable “feeding and water supply” model. As displayed in Table 1, a higher age of piglets at the beginning of pre-starter feeding (in the farrowing area) increased the risk of EDEC presence. The risk was significantly increased for those farms that did not provide a pre-starter to the piglets earlier than day 11 of life.

**Table 1:** Multivariable “feeding and water supply” risk model for EDEC on commercial pig farms in Germany

Risk factor	Categories/Units	OR	*P*-value	LCL	UCL		IRR	P-value	LCL	UCL
Calcium content in feed (2 cat)	1 (≤ 0.70 %)	Baseline					Baseline			
	2 (>0.70 %)	0.327	0.095	0.088	1.214		**0.283**	**0.007**	**0.114**	**0.703**
	Constant	0.765	0.467	0.371	1.574		0.333	0.000	0.212	0.524
Calcium content in feed (2 cat)	1 (< 0.72 %)	Baseline					Baseline			
	2 (≥ 0.72 %)	0.291	0.089	0.070	1.210		**0.227**	**0.005**	**0.081**	**0.635**
	Constant	0.737	0.386	0.369	1.470		0.323	0.000	0.209	0.499
Use of self-produced feed (2 cat)	0 (no)	Baseline					Baseline			
	1 (yes)	2.375	0.053	0.989	5.705		1.612	0.094	0.922	2.817
	Constant	0.421	0.004	0.235	0.755		0.213	0.000	0.147	0.310
Age at pre-starter feeding (4 cat)	1 (≤ 5 days)	Baseline					Baseline			
	2 (6 - 7)	1.250	0.732	0.348	4.486		1.746	0.177	0.778	3.921
	3 (8 - 10)	1.250	0.746	0.324	4.826		1.450	0.402	0.608	3.460
	4 (>10 days)	**4.643**	**0.015**	**1.355**	**15.908**		**2.420**	**0.024**	**1.124**	**5.208**
	Constant	0.400	0.028	0.176	0.908		0.175	0.000	0.101	0.302
Age at pre-starter feeding (2 cat)	0 (< 14 days)	Baseline					Baseline			
	1 (≥ 14 days)	2.804	0.079	0.888	8.860		1.388	0.371	0.677	2.846
	Constant	0.535	0.015	0.322	0.887		0.261	0.000	0.189	0.360
Fiber content in feed (4 cat)	1 (≤ 3.50 %)	Baseline					Baseline			
	2 (3.51 - 3.92)	1.667	0.570	0.286	9.708		0.937	0.909	0.305	2.881
	3 (3.93 - 4.20)	2.083	0.399	0.378	11.482		1.354	0.574	0.471	3.891
	4 (>4.20 %)	2.381	0.325	0.423	13.387		1.500	0.454	0.519	4.331
	Constant	0.300	0.067	0.083	1.090		0.200	0.000	0.093	0.431
Fiber content in feed	Log10 (%)	5.010	0.248	0.326	77.052		1.749	0.155	0.810	3.777
	Constant	0.054	0.134	0.001	2.454		0.105	0.000	0.032	0.340

Multivariable logistic regression (outcome: EDEC farm status, yielding odds ratio) and negative binomial logistic regression (outcome: number of EDEC-positive pens on farm, yielding incidence rate ratio) results with feeding- and water supply-related risk factors identified as candidates in univariable statistics (FET/ANOVA p-value < 0.25). Significant results (p < 0.05) are highlighted in bold.

Abbreviations: *cat *(categories), *EDEC* [Stx2e- and F18 fimbriae-encoding *E. coli* (*E. coli* carrying the genes stx2e and fedA)], *IRR *(Incidence rate ratio), *LCL *(Lower control limit), *OR *(Odds ratio), *UCL *(Upper control limit)

The other variables related to feeding or water supply revealed no significance in the univariable analysis (p-value ≥ 0.25) or were previously excluded from the statistical analysis due to a lack of variability between farms. These included the animals per feeding place ratio, rationed (also number of rations) or ad libitum feeding in the flat deck area, dry or liquid feeding, sensor feeding, automatic feeders (dry or mash), use of milk replacer, use of multiple feeding rations (and their blends) and components contained in the feed for weaned piglets such as soy, milk powder/whey, potato starch and food by-products. Likewise, in the univariable analysis, no significant differences could be found between EDEC-positive and negative farms regarding the water supply of the piglets (number of animals per drinking place, use of public water or wells, bowl drinkers, nipple drinkers or aqua level). Use of additives in feed or water (acids, pre- and probiotics, oligo-/polysaccharides, yeasts, herbs, essential oils, vitamins, and electrolytes) was also tested as a possible risk factor. However, differences between EDEC-positive and EDEC-negative farms were not significant in this respect either. Overall, 55.6% of the farms reported zinc oxide supplementation of piglet feed, 31.3% colistin sulphate supplementation and 39.4% feed supplementation with other antibiotics. For all these variables, there was an almost identical ratio of EDEC-positive to EDEC-negative farms (univariable p-value ≥ 0.44).

To identify further risk factors that may be important for EDEC occurrence during the weaning period, parameters related to the piglet health status were investigated, e.g. current symptoms in weaned piglets and lab-based detection of certain pathogens (STEC, *Escherichia coli*, TGEV, EVDV, Rotavirus, *Clostridium* spp., *Salmonella* spp., *Lawsonia intracellularis*, *Streptococcus* spp., *Glaesserella parasuis*, PCV-2, PRRSV, *Brachyspira hyodysenteriae/pilosicoli*, parasites or others) on the farms in the six months before the sampling of this cross-sectional study. In total, 24 indicators from the questionnaire related to piglet health were again selected according to biological plausibility as candidates for univariable analysis (Additional Table 6). Nine variables with univariable p-value < 0.25 were included in the GLM (Table 2). In this multivariable model the proportion of weaned piglets with dyspnea and the proportion of weaned piglets that suddenly died were significantly higher on EDEC-positive than on EDEC-negative farms, while previous detection of *Streptococcus* spp. in weaned piglets was associated with a lower risk of EDEC-positive pens (Table 2).

**Table 2:** Multivariable “health” risk model for EDEC on commercial pig farms in Germany

Risk factor	Categories/Units	OR	P-value	LCL	UCL	IRR	P-value	LCL	UCL
Proportion piglets with diarrhea (%)	0 (0)	Baseline				Baseline			
	1(0.01 - 1.43)	0.302	0.105	0.071	1.284	0.436	0.086	0.169	1.125
	2 (1.44 - 10.0)	1.783	0.286	0.617	5.155	1.587	0.180	0.808	3.117
	3 (>10.0)	0.436	0.191	0.126	1.511	0.832	0.644	0.381	1.816
	Constant	0.765	0.467	0.371	1.574	0.258	0.000	0.159	0.419
Proportion of piglets with dyspnea (%)	0 (0)	Baseline				Baseline			
	1 (0.01 - 2.0)	**6.337**	**0.002**	**1.996**	**20.115**	**2.143**	**0.021**	**1.124**	**4.088**
	2 (>2.0)	0.849	0.822	0.203	3.555	1.122	0.797	0.467	2.693
	Constant	0.442	0.003	0.258	0.758	0.213	0.000	0.150	0.302
Proportion of piglets with dyspnea (%)	0 (0)	Baseline				Baseline			
	1 (0.01 - 0.50)	**5.658**	**0.049**	**1.007**	**31.800**	2.171	0.116	0.826	5.704
	2 (>0.50)	2.469	0.071	0.926	6.580	1.647	0.115	0.885	3.065
	Constant	0.442	0.003	0.258	0.758	0.213	0.000	0.150	0.302
Proportion of piglets with edema (%)	0 (0)	Baseline				Baseline			
	1 (0.01 - 0.50)	4000	0.126	0.677	23.633	1.989	0.189	0.713	5.553
	2 (>0.50)	1.833	0.221	0.694	4.843	1.397	0.295	0.747	2.610
	Constant	0.500	0.009	0.296	0.844	0.229	0.000	0.163	0.322
Proportion of piglets with edema (%)	0 (0)	Baseline				Baseline			
	1 (>0)	2.143	0.096	0.874	5.256	1.519	0.153	0.857	2.692
	Constant	0.500	0.009	0.296	0.844	0.229	0.000	0.163	0.322
Proportion of piglets that suddenly died (%)	0 (0)	Baseline				Baseline			
	1 (0.01 - 0.50)	3.231	0.055	0.974	10.721	**2.739**	**0.012**	**1.252**	**5.992**
	2 (0.51 - 1.0)	1.909	0.347	0.497	7.337	2.120	0.092	0.885	5.077
	3 (>1.0)	**3.818**	**0.032**	**1.125**	**12.955**	**2.514**	**0.024**	**1.127**	**5.611**
	Constant	0.286	0.007	0.115	0.708	0.129	0.000	0.070	0.237
Proportion of retarded piglets (%)	0 (0)	Baseline				Baseline			
	1 (>0)	2.268	0.150	0.744	6.914	1.669	0.160	0.817	3.411
	Constant	0.333	0.033	0.121	0.917	0.175	0.000	0.091	0.334
Previous lab-based detection of *Escherichia coli*	0 (no)	Baseline				Baseline			
	1 (yes)	1.800	0.184	0.757	4.281	1.111	0.708	0.639	1.932
	Constant	0.444	0.019	0.225	0.877	0.247	0.000	0.162	0.378
Previous lab-based detection of *Streptococcus* spp.	0 (no)	Baseline				Baseline			
	1 (yes)	0.450	0.071	0.189	1.070	**0.531**	**0.029**	**0.301**	**0.936**
	Constant	0.889	0.675	0.513	1.540	0.334	0.000	0.235	0.473

Multivariable logistic regression (outcome: EDEC farm status, yielding odds ratio) and negative binomial logistic regression (outcome: count of EDEC-positive pens on farm, yielding incidence rate ratio) results with selected health-related risk factors identified as candidates in univariable statistics (FET/ANOVA p-value < 0.25). Significant results (p < 0.05) are highlighted in bold.

Abbreviations: *cat* (categories), *EDEC *[Stx2e- and F18 fimbriae-encoding *E. coli *(*E. coli* carrying the genes stx2e and fedA)], *IRR* (Incidence rate ratio), *LCL* (Lower control limit), *OR *(Odds ratio), * UCL* (Upper control limit)

Of the management variables recorded in the questionnaires, 22 were considered as biologically plausible risk factors for analysis in univariable models (Additional Table 7). Based on the results, seven of these variables were selected as candidates for subsequent GLM analysis in which four yielded significant associations (Table 3). In the multivariable models a weaning age between 26 and 28 days was associated with a significantly higher risk of EDEC presence. A weaning age above 28 days resulted in a lower risk in the multivariable logistic regression but without significance (Table 3). Routine vaccination of sows with antigens of *Clostridium* spp. significantly reduced the risk of EDEC presence while vaccination of sows and/or piglets against *Lawsonia intracellularis* as well as vaccination of piglets against STEC were significantly associated with EDEC presence on the farm (Table 3). All other variables in the area of hygiene – which included the separation of piglets according to weight, age, sex, or vaccination status, different or same origin of piglets, all in – all out procedure, recommended cleaning steps and use of a cleaning agent, disinfection and exposure times of the disinfectant, drying and vacancy of the barn after disinfection, concentration check of the disinfectant, use of a high-pressure cleaner, use of the farm’s own clothing and compulsory showering before entering the barn – showed no relevant association in the univariable analysis (*p* > 0.25) or were previously excluded from the statistical analysis due to a lack of variability between farms. This also applied to the parameters of stable climate (ambient temperature, draught, microclimate zones and bedding/straw, floor design, ventilation technology).

**Table 3:** Multivariable “management” risk model for EDEC on commercial pig farms in Germany

Risk factor	Categories/Units	OR	P-value	LCL	UCL		IRR	P-value	LCL	UCL
*Clostridium* spp. vaccination of sows	0 (no)	Baseline					Baseline			
	1 (yes)	**0.417**	**0.047**	**0.176**	**0.989**		**0.486**	**0.011**	**0.279**	**0.848**
	Constant	1.000	1.000	0.538	1.859		0.376	0.000	0.255	0.554
*Lawsonia intracellularis* vaccination of sows and/or piglets	0 (no)	Baseline					Baseline			
	1 (yes)	**2.725**	**0.049**	**1.004**	**7.390**		1.550	0.170	0.829	2.895
	Constant	0.489	0.005	0.297	0.806		0.237	0.000	0.172	0.326
*Mycoplasma hyopneumoniae* vaccination of weaned piglets	0 (no)	Baseline					Baseline			
	1 (yes)	0.141	0.086	0.015	1.317		0.599	0.367	0.196	1.825
	Constant	4.000	0.215	0.447	35.788		0.430	0.125	0.146	1.263
*Mycoplasma hyopneumoniae* vaccination of suckling piglets	0 (no)	Baseline					Baseline			
	1 (yes)	0.479	0.158	0.172	1.331		0.655	0.200	0.342	1.251
	Constant	1.111	0.819	0.451	2.734		0.368	0.001	0.209	0.648
ED vaccination of piglets	0 (no)	Baseline					Baseline			
	1 (yes)	**3.333**	**0.035**	**1.087**	**10.218**		1.794	0.091	0.912	3.530
	Constant	0.500	0.005	0.309	0.808		0.234	0.000	0.172	0.320
Herd specific vaccination (2 cat)	0 (no)	Baseline					Baseline			
	1 (yes)	1.765	0.193	0.750	4.152		1.279	0.381	0.737	2.219
	Constant	0.486	0.015	0.272	0.867		0.238	0.000	0.164	0.346
Weaning age of piglets (3 cat)	0 (21 - 25 days)	Baseline					Baseline			
	1 (26 - 28 days)	**4.400**	**0.003**	**1.666**	**11.619**		**2.733**	**0.002**	**1.468**	**5.089**
	2 (>28 days)	0.882	0.842	0.259	3.011		1.240	0.589	0.568	2.704
	Constant	0.333	0.003	0.163	0.682		0.146	0.000	0.091	0.236

Multivariable logistic regression (outcome: EDEC farm status, yielding odds ratio) and negative binomial logistic regression (outcome: count of EDEC-positive pens on farm, yielding incidence rate ratio) results with selected management-related risk factors identified as candidates in univariable statistics (FET/ANOVA p-value < 0.25) Significant results (p < 0.05) are highlighted in bold.

Abbreviations: *cat* (categories), *EDEC* [Stx2e- and F18 fimbriae-encoding *E. coli* (*E. coli *carrying the genes stx2e and fedA)], *ED* (Edema disease), *IRR* (Incidence rate ratio), *LCL* (Lower control limit), *OR* (Odds ratio), *UCL* (Upper control limit)

Twenty-four farm structure and performance variables were identified as biological plausible candidates for the univariable models, of which two were selected as candidates (univariable p-value < 0.25) for the GLM analysis (Additional Table 8). Here, a higher weight of piglets at weaning (> 6 kg) as well as a higher piglet weight at the end of the flat deck period (> 28 kg) were significantly associated with EDEC presence (Table 4). Further variables that were previously excluded from the statistical analysis due to a lack of variability between farms or without significance in the univariable analysis from this group were conventional or organic husbandry of the pigs, different production types (piglet producer, rearing farm, fattening farm, or closed system), additional purchase of pigs, farm size, production rhythm, number of weaned piglets per run and overcrowding in the flat deck area. Other performance parameters (number of weaned piglets/litter, housing days in the flat deck area and average losses in the flat deck sector) also showed no relevant association in the univariable analysis or were excluded from further statistical analysis for the reasons mentioned above.

**Table 4:** Multivariable ***“***farm structure and performance***”*** risk model for EDEC on commercial pig farms in Germany

Risk factor	Categories/Units	OR	P-value	LCL	UCL		IRR	P-value	LCL	UCL
Starting weight of piglets at flat deck period (kg)	0 (≤ 6.0)	Baseline					Baseline			
	1 (6.1 - 7.0)	**3.667**	**0.049**	**1.008**	**13.343**		1.913	0.130	0.827	4.428
	2 (7.1 - 7.9)	2.895	0.065	0.937	8.946		**2.493**	**0.012**	**1.221**	**5.089**
	3 (>7.9)	4.400	0.051	0.991	19.545		2.247	0.096	0.867	5.824
	Constant	0.273	0.005	0.111	0.673		0.143	0.000	0.080	0.254
Piglet weight at end of flat deck period (kg)	0 (< 28)	Baseline					Baseline			
	1 (28 - 28.9)	1.167	0.812	0.327	4.159		1.084	0.853	0.464	2.531
	2 (29 - 30.9)	**4.875**	**0.015**	**1.360**	**17.472**		**2.696**	**0.016**	**1.203**	**6.042**
	3 (≥ 31)	2.500	0.150	0.718	8.710		**2.553**	**0.022**	**1.143**	**5.702**
	Constant	0.333	0.020	0.132	0.840		0.150	0.000	0.081	0.276

Multivariable logistic regression (outcome: EDEC farm status, yielding odds ratio) and negative binomial logistic regression (outcome: count of EDEC-positive pens on farm, yielding incidence rate ratio) results with selected farm structure and performance-related risk factors identified as candidates in univariable statistics (FET/ANOVA p-value < 0.25). Significant results (p < 0.05) are highlighted in bold.

Abbreviations: *cat* (categories), *EDEC* [Stx2e- and F18 fimbriae-encoding *E. coli* (*E. coli *carrying the genes stx2e and fedA)], *IRR* (Incidence rate ratio), *LCL* (Lower control limit), *OR* (Odds ratio), *UCL* (Upper control limit)

## Discussion

In the previously published first part of our cross-sectional study, we were able to determine an EDEC prevalence of 37.4% of German pig farms [1]. A questionnaire applied through an on-site interview with the farm manager during the farm visits was used to collect information on possible risk factors associated with the farm level EDEC status. The questionnaire included various parameters and was divided into the four domains feeding and water supply, health, management, and farm structure and performance. Based on current literature, risk factors for the occurrence of edema disease in piglets are mainly related to feeding strategies [24, 38–40] genetic susceptibility of the animals [41–43] and several deficiencies or stressors that may be present during the weaning period, e.g. loss of lactogenic protection, insufficient passive and active immunization, as well as change in housing conditions or management practices [9, 44–47]. Most of the latter studies refer to general bacterial infections or to the closely related post weaning diarrhea (PWD) of weaned piglets, maybe some of those findings can also be applied to EDEC detection. However, to the best of our knowledge, our study is the first systematic investigation under field conditions aimed at identifying risk factors for the presence of EDEC, used here as a proxy for edema disease in weaned piglets. As mentioned in the first published part of this cross-sectional study [1], we are aware that EDEC detection in saliva-soaked ropes or floor samples cannot be equated with evidence of EDEC infection or even clinical edema disease. Nevertheless, the lab-based detection of EDEC in those samples was seen as the most suitable outcome to investigate risk factors of edema disease, as focusing on clinical symptoms would have been too imprecise due to the considerable number of differential diagnoses for ED [1]. Another benefit was the reduction of stress for animals during sampling and the costs for laboratory tests remained within reasonable limits.

As highlighted in the first part of this study, we are aware that the farms in this study were not randomly selected as we did not have access to population data. After all, the demographic characteristics of the study farms suggest that they well represented the German pig farming production system [48]. As referred before, we cannot rule out the possibility that some farms participated in the study because they struggled with health issues in the nurseries. However, we had no evidence for such bias from the farm visit records (e.g., vaccination against edema disease and history of laboratory detection of STEC in the past).

Not all the variables collected in the survey and considered biologically relevant in the context of EDEC risk could be further assessed in the uni- and multivariable regression models. The main reasons for this were item-based missing values that reduced the available sample size to less than half of the farms, and insufficient variability with zero frequencies in some variable categories. Of those variables available for the risk factor models, several were significantly associated with farm EDEC status, either in the logistic regression or in the negative binomial model, or in both models.

From the domain “feeding and water supply” it was most interesting that both models showed a significantly increased risk of EDEC presence when the pre-starter was offered only to piglets older than ten days of age. This result agrees with findings of others that early adaptation of the gastrointestinal tract of piglets to a solid diet via supplementary creep feeding during the nursing period can prevent pathological proliferation of microorganisms in the gut, although there are different results on the influence of pre-starter feeding. Already in a study from 1984, Miller et al. concluded that creep feeding led to reduced diarrhea after weaning but worsened the symptoms if the solid feed was only consumed in small quantities (< 100 g solid feed) before weaning [49]. Bruininx et al. demonstrated that creep feeding promoted earlier feed intake and better performance after weaning, as measured by weight gain [39]. In 2005, Carstensen et al. investigated more specifically the effect of offering supplementary creep feed to suckling piglets (second to fourth week of life) on the fecal shedding of hemolytic *E. coli* bacteria after weaning and occurrence of PWD [50]. In that study the occurrence of diarrhea and fecal shedding of hemolytic *E. coli* O149 was not associated with creep feeding per se. However, the fecal shedding of that bacteria occurred significantly less often in piglets that had shown only slight interest in the offered creep feed (i.e. contacted the feed less frequently than or equal to the median level of contact) compared to those piglets that contacted offered creep feed more frequently or to other piglets that had no access to creep feed at all [50]. The authors also observed that the former piglets consumed less feed mass during the first two days after weaning and suspected that this reduction could have led to a lower intestinal proliferation of *E. coli* bacteria after weaning [50]. On the other hand, in some earlier reports, creep feeding did not reveal any effect on the health status of weaned piglets [51, 52] or even triggered the onset of PWD under experimental conditions [53]. A review from 2002 also compiled data on the effects of creep feeding on PWD [54]. The results were inconsistent, and when positive effects were found, the optimal creep feed intake (high, moderate, or low) was not clear [54]. In a more general perspective, Madec et al. detected that low feed intake during the first week after weaning was associated with a higher risk of gastrointestinal disease in piglets [44]. McCracken et al. showed that insufficient feed intake during the immediate postweaning period can lead to inflammation in the small intestine and compromises villus-crypt structure and function [45]. Considering the hypothesis that avoiding anorexia or very low food intake after weaning reduces the risk of digestive disorders, supplementary creep feeding is recommended [54], and the results of our study indicate that it should be started before 10 days of the piglets’ life to reduce the risk of EDEC occurrence.

Since there is some promising data that use of additives in feed or drinking water (acids, pre- and probiotics, oligo-/polysaccharides, yeasts, herbs, essential oils, vitamins and electrolytes) can improve the intestinal health of pigs [47, 55–60], we investigated some supplements as risk factors for EDEC presence in pens of weaned piglets in this study. However, we could not find any differences between EDEC-positive and EDEC-negative farms in this respect, which also applied to the use of zinc oxide, colistin sulphate or other antibiotics in feed or drinking water. Based on these results, in our study farms there was no evidence that these additives or medications provided significant protection against EDEC presence. This contrasts with other studies where they were shown to affect the prevalence of EDEC infections or the incidence of ED outbreaks among piglets [25, 29, 37, 61–64]. Considering the current antimicrobial resistance situation in STEC and the adverse effects of zinc oxide on the development of such resistance [23, 29–32], our study suggests that the use must also be viewed critically in terms of pro- or metaphylactic efficacy against ED. We would like to point out that the samples for our study were taken before the EU regulation banning the use of ZnO in therapeutic doses in 2022 [65].

Some pig breeds have a genetically determined resistance to mucosal colonization by those *E. coli* strains that express F18 fimbriae [42]. We therefore tried to evaluate breed as a determinant for EDEC presence as well. Unfortunately, our attempts failed. The participating farms indicated 50 different breeds or hybrid crosses and often used various boars for breeding that breed as a potential risk factor eluded our statistical analyses.

Our survey covered several variables relating to health status of the piglets. For this purpose, data was requested from each farm about laboratory-based detection of various infectious agents in weaned piglets within the last 6 months before the survey was started. In addition, all vaccinations conducted on the farm (and/or farm of origin of the piglets) were noted. Some variables in this domain proved to be significantly associated with the presence of EDEC. Among others, the proportions of piglets with dyspnea and of sudden deaths were higher in EDEC -positive than in -negative farms. On the one hand, this finding may indicate that a generally poorer state of health is a risk for EDEC presence, but on the other hand it may imply that EDEC infections lead to an increased incidence of these symptoms. In this case, the association, although significant, gives no indication of what is cause und what is effect. We also found that farms had a lower risk of EDEC presence when *Streptococcus* spp. had been detected there before according to questionnaire entries. Due to the cross-sectional nature of this study, no chronological sequence could be disclosed in this study. Therefore, improvement of the hygiene conditions on the farms, possibly initiated because of *Streptococcus* spp. detection in the past, could have had the side effect that EDEC infections occurred less frequently. However, we could not identify significant risk factors among the hygiene related parameters tested such as mixing of piglets into groups, different origin of piglets, cleaning and disinfection and biosecurity measures. Vaccination of sows against *Clostridium* spp. also resulted in a lower risk of EDEC presence. All clostridial vaccines licensed for use in pigs in Germany contain antigens of *Clostridium perfringens* type C and/or *Clostridium perfringens* type A and *Clostridioides difficile* [66]. Types A and C of *C. perfringens* and *Clostridioides difficile* are enteric pathogens of swine and can cause neonatal diarrhea in this species [67], with a pathogenesis that includes damage to the epithelial barrier of the small or large intestinal mucosa, respectively [67, 68]. Vaccination of sows leads to subsequent passive immunoprotection of their piglets against these enteric pathogens [69]. It seems conceivable that this specific immunization generally improved the gastrointestinal health of piglets of this study and protected non-specifically to some degree against infection with EDEC. In contrast, both vaccination against *Lawsonia intracellularis* and vaccination against STEC, the latter being equivalent to the administration of licensed Stx2e toxoid vaccines, appeared in this study as higher risks for the farms to be affected by EDEC presence. *Lawsonia intracellularis* is a gastrointestinal bacterial pathogen of pigs that is widespread throughout Europe and beyond [70]. Although there are many unresolved issues in *L. intracellularis* infection, it is thought that this pathogen affects the microbiome of pigs [71]. It appears reasonable to assume that farms that are forced to deal with several gastrointestinal pathogens vaccinate against *L. intracellularis* more frequently to improve the health status of their piglets. Similarly, the positive association between STEC vaccination and EDEC presence may simply reflect that those farms being positive for this pathogen had an issue with ED and, thus, administered respective vaccines more often. However, use of these vaccines cannot reduce the prevalence of EDEC since only Stx2e toxoid vaccines are available, and these vaccines mediate protection by inducing Stx2e-neutralizing antibodies but do not prevent infection or multiplication of EDEC bacteria in vaccinated piglets [21, 22].

Early weaning (weaning at two to four weeks of age) is well documented as stressful and associated with an increased incidence of diseases [44, 72–74]. In most current swine breeding systems, weaning is a sudden event that puts the piglets under significant physical and psychological stress [75, 76] thus promoting clinical manifestation of ED [9, 13] or other syndromes in these piglets in the following days [44, 46, 77]. According to current EU legislation, no piglets shall be weaned from the sow at less than 28 days of age unless the welfare or health of the dam or the piglet would otherwise be adversely affected [78]. However, piglets may be weaned up to seven days earlier if they are moved into specialized housings which are emptied and thoroughly cleaned and disinfected before the introduction of a new group, and which are separated from housings where sows are kept, in order to minimize the transmission of diseases to the piglets [78]. In our data set, the arithmetic mean and median for the weaning age of piglets was 25.6 and 26 days, respectively, which is within the range of the exemption for early weaning under 28 days. However, results of our study were inconclusive in this respect and difficult to interpret. Both the multivariable logistic regression and the negative binomial regression revealed that farms weaning at 26–28 days of age had a significantly increased risk of EDEC presence over farms that weaned earlier. On farms that weaned later (above 28 days) the risk of EDEC presence was higher in the negative binomial regression than the baseline (weaning age 21–25 days) but not significantly, and much lower than on the former farms. In the multivariable logistic regression, a weaning age above 28 days indicated the lowest risk for EDEC presence, but also without significance. We must emphasize here that only three of the studied farms weaned their piglets after the 28th day of life, a larger sample size might have yielded a different value for significance. Due to the epitheliochorial placenta of pigs, piglets have an incomplete immune system until they are approximately 4 weeks old and are dependent on the transmission of maternal immunity via colostrum and milk [79, 80]. Therefore, it has been suggested that the delay in the onset of ED until after weaning is due to the decline of maternal immunity during the lactation period [81]. This could be consistent with our findings that younger piglets still benefit from protection provided by maternal antibodies, while older piglets experience an immunological gap until their immune system is fully developed. In the study from Vidal et al. the immunogenicity of a vaccine candidate against STEC was evaluated: Here, all experimental groups showed a basal concentration of specific IgG and IgA antibodies, which could be increased by vaccination in the experimental groups compared to the control group [81]. There was an effective passive transfer of specific antibodies from immunized sows to their offspring, with antibody levels remaining relatively high from days 15 to 30 post-birth [81]. However, a slight decrease reflected a progressive decline in maternal protection as the piglets aged [81]. The authors clarify that the long-term persistence of these specific antibodies in piglets is uncertain, and further active immunization may be necessary [81].

Unfavorable housing conditions and management practices before, at and after weaning are frequently mentioned in the literature as imposing stressors for the piglets in addition to weaning per se [82–84]. However, apart from the distinct vaccinations mentioned above, none of the analyzed variables related to management (e.g., stable climate or hygiene measures) was significantly associated with the EDEC farm status determined in our study.

Among the variables related to farm structure and performance, the body weights at the beginning and end of the flat deck period presented clearly as risk factors for EDEC occurrence at farm level in our analysis. Here, there was a higher risk for farms with heavier animals, although not all odds ratios or incidence rate ratios, respectively, were significant. These findings are consistent with empirical evidence and experimental data that intensive and nutrient rich feeding stimulates the intestinal proliferation of EDEC and triggers development of colienterotoxemia (ED) in piglets. Smith and Halls investigated the effect of nutrition in an oral infection model of ED and PWD. They observed that a reduction in the amount of feed or a diet with an unusually low content of nutrients significantly lowered the bacterial number of the inoculated *E. coli* strain in rectal contents of the piglets and prohibited the clinical onset of diarrhea, anorexia, and ED [85]. In a similar model, but with many more piglets, Bertschinger et al. showed that a weaner diet extremely low in nutrients (5% crude protein, 4.6 MJ/kg digestible energy, 17% crude fiber) completely prevented the colienterotoxemia (ED) by inhibiting intestinal proliferation of the pathogenic *E. coli* O139:K82(B): H4 strain that was orally administered to the weaned piglets [86]. After replacement of the poor diet by commercial feed, the bacterial numbers of the inoculated *E. coli* strain in the feces increased, and pigs were affected by fatal colienterotoxemia [87]​. Bosworth et al. established an infection model using a defined EDEC strain for oral infection of weaned piglets fed with a commercial feed containing either 17%, 18% or 21% crude protein. No clinical signs of ED were observed in any piglet of both former groups but microangiopathy consistent with subclinical ED was histologically evident in 38% and 50% of the piglets, respectively. In contrast, 59% of the piglets fed with 21% protein developed ED and 94% showed microangiopathy [88]. Evidence compiled in the review from Kim et al. also indicates that infectious gastrointestinal diseases like PWD are associated with dietary components​ [38]. It therefore appears reasonable to assume that the association of high body weight and EDEC presence in our study reflects nutrient-rich feeding of the weaned piglets. However, neither the protein content nor the fiber content in the weaner feed ration were identifiable as a risk factor for EDEC presence in our data. We therefore suspect that it was primarily the non-restricted supply (*ad libitum* feeding) of highly concentrated feed rations in some farms that stimulated fecal shedding of EDEC thus increasing environmental EDEC contamination of the pens. Unfortunately, the daily feed intake was not reported by the farms and could therefore not be analyzed here. In any case, our results indicate that farms with excellent weight gains of their piglets can be particularly exposed to the ED pathogen.

## Conclusions

Although most of the parameters examined in the questionnaires were already excluded in the univariable analysis due to lack of significance, we were able to find risk factors for the occurrence of EDEC on German pig farms in all four multivariable models. The results of our study suggest that feeding a pre-starter to piglets before 10 days of life may be useful to reduce the risk of EDEC detection. Whether there is an increased risk for farms dealing with respiratory diseases in weaned piglets or whether an increase in respiratory symptoms is more likely a result of infection with EDEC may be the subject of further investigations. Based on our study, we cannot conclusively explain the influence of weaning age on EDEC detection. However, since only three of the studied farms weaned their piglets after the 28th day of life, a larger sample size might have yielded a different value for significance. In our study, farms that weaned before the 26th day of life seemed to have a lower risk for EDEC detection, even if the reasons for this interrelation could not be clarified by our study. Due to the study design, we can only speculate whether vaccination of sows against *Clostridium* spp. is beneficial for farms in terms of reducing the risk of EDEC detection.

## Supplementary Information

Additional file 1. Questionnaire project “STEC prevalence”. Complete questionnaire used for data collection (in English).

Additional file 2. Additional Table 1: Descriptive information on participating farms regarding the farm structure. Additional Table 2: Descriptive information on participating farms regarding the health status. Additional Table 3: Descriptive information on participating farms regarding the performance. Additional Table 4: Descriptive information on participating farms regarding the clinical signs in weaned piglets

Additional file 3. Additional Table 5: Explorative univariable association between EDEC farm status and farm level feeding and water supply variables. Additional Table 6: Explorative univariable association between EDEC farm status and farm level health-related variables. Additional Table 7: Explorative univariable association between EDEC farm status and farm level management variables. Additional Table 8: Explorative univariable association between EDEC farm status and farm level production and performance variables

## Data Availability

The datasets used and/or analyzed during the current study are available from the corresponding author on reasonable request. The English translation of the questionnaire can be found in the supplementary material **(Additional file 1)**.

## Abbreviations

ANOVA: One-way analysis of variance
Cat: Categories
CNS: Central nervous system
DNA: Deoxyribonucleic acid
*E. coli*: *Escherichia coli*
ED: Edema disease
EDEC: Stx2e- and F18 fimbriae-encoding *E. coli (E. coli *carrying the genes *stx2e *and* fedA)*
EVDV: Epidemic virus diarrhea virus
*fedA*: Gene encoding for the major protein subunit of F18 fimbriae
FET: Fisher´s exact test
GLM: Generalized linear models
IRR: Incidence rate ratio
LCL: Lower control limit
OR: Odds ratio
PCR: Polymerase chain reaction
PCV-2: Porcine Circovirus 2
PPV: Porcine Parvovirus
PRRSV: Porcine reproductive and respiratory syndrome virus
PWD: Post weaning diarrhea
STEC: Shiga toxin-encoding/-producing *E. coli*
*stx2e*: Gene encoding for the Shiga toxin subtype Stx2e
Stx2e: Shiga toxin subtype Stx2e
TGEV: Transmissible gastroenteritis virus
UCL: Upper control limit
