# Supplementary material for: Cross-sectional survey of risk factors for edema disease Escherichia coli (EDEC) on commercial pig farms in Germany
Source: BMC Vet Res. 2025 Oct 3;21:576. doi: 10.1186/s12917-025-05054-7 (PMC12492929; doi:10.1186/s12917-025-05054-7)
Supplement: Supplementary file 1 — Additional file 1. Questionnaire project “STEC prevalence”. Complete questionnaire used for data collection (in English). [file 12917_2025_5054_MOESM1_ESM.pdf]

Questionnaire Project "STEC Prevalence"

1 Information on the farm structure
Please select all applicable answers!

1.1 Type of business
☐ Conventional husbandry ☐ Organic farm

☐ Other (e.g. Animal Welfare Initiative): ....

1.2 Production Type
☐ Piglet producers ☐ Rearing farm

☐ Rearing and fattening ☐ Closed system

☐ Other:..................................

Purchase of: ☐ Gilts ☐ Piglets☐ Fattening pigs

Breed(s) kept (genetics): ....

1.3 Farm size (current number of animals)

Sows	
Weaned piglets (flat deck)	
Fattening pigs	

1.4 Occupancy on the flat deck

How often are new piglets housed in the flat deck (production rhythm)?	
Number of newly housed piglets per run	

☐ No overcrowding ☐ Partial overcrowding ☐ Significant overcrowding

1.5 Performance parameters:

Parameter	Average (in the last 6 months)
Number of weaned piglets/litter	
Weaning age [days]	
Starting weight flat deck [kg]	
End weight flat deck [kg]	
Housing days in the flat deck	
Losses in the flat deck [%]	
Feed conversion in the flat deck [1:x]	

2 Health status of weaned piglets

2.1 Mortality and morbidity in the last 3 rounds

Round	Mortality (losses in %)	Morbidity (diseased animals in %)
Actual round		
Last round		
Penultimate round		

2.2 Do weaned piglets currently experience the following symptoms?

Symptom	Number of weaned piglets affected [%]
Diarrhea	
Retarded	
Dyspnea	
CNS symptoms	
Edema	
sudden death	

Other:...

☐ It is a recurring problem on this farm

2.3 Have the following pathogens been detected in laboratory diagnostics in the weaned piglets within the last 6 months?

Please select all applicable answers!

☐ Shigatoxin producing *E. coli* (STEC) ☐ other *E. coli* ☐ TGEV

☐ EVDV ☐ Rotavirus ☐ *Clostridium* spp.

☐ *Salmonella* spp. ☐ *Lawsonia intracellularis* ☐ Parasites

☐ *Streptococcus* spp. ☐ *Glaesserella parasuis* ☐ PCV-2

☐ PRRSV ☐ *Brachyspira hyodysenteriae/ pilosicoli*

☐ Other:..

2.4 Vaccinations used in the herd of origin and for weaned piglets

Vaccination against...	Sows	Suckling piglets	Weaning piglets	Farm is unsuspicious ("free")
Edema disease (STEC)	☐	☐	☐	☐
E. coli diarrhea	☐	☐	☐	☐
Circovirus (PCV-2)	☐	☐	☐	☐
Clostridium spp.	☐	☐	☐	☐
Lawsonia intracellularis (ileitis)	☐	☐	☐	☐
Salmonella spp.	☐	☐	☐	☐
PRRS	☐	☐	☐	☐
Mycoplasma hyopneumoniae	☐	☐	☐	☐

Glaeserella parasuis	☐	☐	☐	☐
APP	☐	☐	☐	☐
Rhinitis atrophicans	☐	☐	☐	☐
Erysipelas rhusiopathiae	☐	☐	☐	☐
Parvovirus	☐	☐	☐	☐
Leptospirosis	☐	☐	☐	☐
Influenza	☐	☐	☐	☐
Herd-specific vaccine against ...				
	☐	☐	☐	
	☐	☐	☐	
	☐	☐	☐	

3 Hygiene management

3.1 Measures to be taken when weaning piglets

Please select all applicable answers!

☐ Separation into weight groups (max. 3kg difference)	☐ in compartments	☐ into pens
☐ Separation into age groups (max. 1 week difference)	☐ in compartments	☐ into pens
☐ Separation by gender	☐ in compartments	☐ into pens
☐ Separation of vaccinated/non-vaccinated animals	☐ in compartments	☐ into pens

3.2 Origin of weaned piglets

☐ from different farms of origin ☐ currently from the same farm of origin

☐ always from the same farm of origin

3.3 Cleaning and disinfection in the sampled flat deck

Please select all applicable answers!

☐ Continuous occupancy **or** In-Out Procedure: ☐ Compartment ☐ whole barn

3.3.1 Please select all the steps that are carried out during cleaning:

☐ 1. Rough cleaning ☐ 2. Soaking ☐ 3. Wet cleaning ☐ 4. Drying

☐ The given order (1. – 4.) is followed

☐ A cleaning agent is used

3.3.2 Please select all the answers that apply to disinfection:

☐ Disinfection after cleaning

☐ Exposure times (according to the manufacturer) are respected

☐ Thorough rinsing ☐ Drying

☐ Vacancy for a few days ☐ Concentration check of the disinfectant

High-pressure cleaner is used for: ☐ cleaning ☐ disinfection

3.4 Biosecurity

☐ Use of farms' own clothing

☐ Compulsory showering before entering the barn

4 Stable climate of the sampled animals in the flat-deck

Please select all applicable answers!

4.1 Ambient temperature

☐ Temperature 26-30°C ☐ Temperature 21-26°C ☐ Temperature <21°C

☐ Noticeable draught ☐Outdoor enclosure ☐ Climate computers

4.2 Floor design

☐ Plastic slatted floor ☐ Concrete slatted floor ☐ Metal slatted floor

☐ Straw/bedding ☐ Open pen walls

☐ Microclimate areas available (e.g. fixed lounging area, cover, underfloor heating..)

4.3 Ventilation technology

☐ Underfloor ventilation ☐ Negative pressure ventilation with fans

☐ Pore/trickle ceiling ☐ Door aisle ventilation

5 Feeding and watering of weaned piglets

5.1 Supplementary feeding before weaning

☐ unknown ☐ no ☐ yes (if yes, please indicate the start and type of food)

➔ offered at day of life: ....

➔ Feed:...

5.2 Feeding technique in the sampled flat-deck

Please select all applicable answers!

Animal-to-feeding place ratio [1: X]: ....

☐ ad libitum ☐ Rationed …………. rations/ day

☐ dry ☐ liquid

☐ self-mixed ☐ conventional-purchasable mixture

☐ Sensor feeding ☐ Longitudinal troughs

☐ Dry Feeder ☐ Automatic mash feeder

☐ Pre-starter ☐ Milk replacer

☐ Rearing feed I and II used ☐ Blended feed

☐ contains soy

☐ contains milk powder/whey

☐ contains potato starch

☐ contains food by-products

5.3 Current composition of the feed for weaned piglets

Dry matter (%)	
Crude protein (%)	
Crude fiber (%)	
Crude fat (%)	
Crude ash (%)	
Calcium (%)	
Sodium (%)	
Phosphorus (%)	
Lysine (%)	
Methionine (%)	
Energy (MJ ME/kg)	

These are:

☐ the declaration of the feed manufacturer

☐ Data from feed analysis

☐ Estimated values

You are welcome to give this data as a copy

5.4 Water supply for weaned piglets

Please select all applicable answers!

Number of piglets per watering place ratio [X:1]: ....

☐ Public water

☐ farm's own well

☐ Bowl drinkers

☐ Nipple drinkers

☐ Chlorinated water

☐ Aqualevel

5.5 Are the following substances used in the feed and/or water of the weaned piglets?

Please select all applicable answers!

	Product and dosage
☐ Colistin (-sulphate)	
☐ other antibiotics	
☐ Zinc (-oxide)	
☐ Organic acids/their salts (formic acid, fumaric acid, potassium diformate, etc.)	
☐ Prebiotics and probiotics	
☐ Oligo-/polysaccharides (e.g. inulin, lactulose...)	
☐ Yeasts	
☐ Herbs/ Essential Oils	
☐ Vitamins/ Electrolytes	

☐ Other	
